# Imperfect Enamel

**Published:** 1869-02

**Authors:** S. P. Cutler


### IMPERFECT ENAMEL.

BY S. P. CUTLER, M. D., D. D. S.

Furrowed enamel is not always an indication of bad taint in blood of an hereditary nature. Not long since a young lady called on me to have her teeth examined.

There were some few decays, with this exception all of her teeth were perfect, except the lower front and lateral incisor of the left side, which were both cross-furrowed deep into the enamel some three or four grooves in each, and otherwise not well developed. She informed me that two corresponding deciduous teeth were knocked out when she was about four years old. This statement settled the rising suspicions in my mind. The high respectability of the party should of itself have done away with all doubts, even without any knowledge of the case.

The above case goes to establish beyond any reasonable doubt the connection existing between the first and second set of corresponding teeth, showing the importance of retaining the deciduous teeth up to the eruptive stage of their respective successors, and that too, in a healthy condition.

The above case shows conclusively that the nutrition of the crowns, especially the enamel, was disturbed to a considerable extent by the accidental loss of the deciduous teeth at too early a stage; had they been retained two years longer, no defects could have existed in the two permanent teeth referred to.

The importance of not only retaining the deciduous teeth up to the full period when nature makes provision for their leaving, by root absorption, as well as their healthy preservation, can not be too highly estimated.

---

CINCINNATI, March 8th, 1869.

*To the Editors of the Dental Register.*

GENTLEMEN: Owing to the fact that a majority of the Junior students were not present at the Commencement Exercises of the Ohio Dental College, I beg leave through your columns, to offer the thanks of the Southern students, to the Faculty and Northern students, for the kind nessand uniform politeness extended to us during the past session.

We trust that in future all similar associations between Northern and Southern men may prove as agreeable.

Very respectfully, Your obedient servant,

JAS. B. ASKEW,

*Committee of One.*

